# Lentivirus transduced interleukin-1 receptor antagonist gene expression in murine bone marrow-derived mesenchymal stem cells *in vitro*

**DOI:** 10.3892/mmr.2015.4003

**Published:** 2015-06-26

**Authors:** TAO HE, GUANGHAO CHI, BO TIAN, TINGTING TANG, KERONG DAI

**Affiliations:** 1 Department of Orthopedics, Shanghai Key Laboratory of Orthopedic Implants, Shanghai Ninth People's Hospital, Shanghai Jiao Tong University School of Medicine, Shanghai 200011, P.R. China; 2 Department of Orthopedics, Changhai Hospital Affiliated to the Second Military Medical University, Shanghai 200433, P.R. China; 3 Department of Orthopedics, Han Zhong Central Hospital, Hanzhong, Shanxi 723000; P.R. China; 4 The Key Laboratory of Stem Cell Biology, Institute of Health Sciences, Shanghai Jiao Tong University School of Medicine and Shanghai Institutes for Biological Sciences, Chinese Academy of Sciences, Shanghai 200025, P.R. China

**Keywords:** murine, mesenchymal stem cell, interleukin 1 receptor antagonist, gene, lentivirus, transfection

## Abstract

Genetically modified mesenchymal stem cells have been used in attempts to increase the expression of interleukin-1 receptor antagonist (IL-1Ra); however, the attempts thus far have been unsuccessful. The aim of the present study was to investigate whether the lentivirus transduced IL-1Ra gene was able to be stably expressed in murine bone marrow-derived mesenchymal stem cells (mBMSCs) *in vitro*. In the present study, third generation lentiviral (Lv) vectors transducing the IL-1Ra/green fluorescent protein (copGFP) gene were constructed and transfected into mBMSCs to establish the Lv.IL-1Ra.copGFP/mBMSCs, which were evaluated using fluorescence microscopy, flow cytometry, cell viability analysis using a cell counting kit-8 kit, Trypan blue staining and an MTT growth kinetics assay. The expression of IL-1Ra was analyzed using reverse transcription-quantitative polymerase chain reaction and western blotting. The results demonstrated that the Lv.IL-1Ra/copGFP vector was successfully constructed. The mBMSCs exhibited a short proliferation life, however they had good growth kinetics at an early stage and improved viability following efficient transduction of the IL-1Ra gene. IL-1Ra was overexpressed following transfection of mBMSCs. In conclusion, lentiviral vector transduced mBMSCs were able to efficiently express exogenous Il-1Ra under certain conditions and had a marked capacity for proliferation.

## Introduction

Mesenchymal stem cells (MSCs) are of great importance in the field of cell therapy. MSCs are multipotent self-renewing cells, which can be easily isolated from bone marrow. Bone marrow-derived MSCs (BMSCs) are capable of differentiation into cells of different lineages (1). MSCs have potential for use in various disease models due to their involvement in tissue repair, their injury site homing abilities following systemic delivery and their evasion of normal host immune responses. Therefore, MSCs are currently considered the most promising adult cell type for therapeutic applications, particularly in regenerative medicine (2).

Interleukin-1 receptor antagonist (IL-1Ra) is a naturally occurring anti-inflammatory protein; it competitively blocks the binding of IL-1α and IL-1β to IL-1 type receptors (3). IL-1Ra is a promising agent used to cure a number of inflammatory and orthopedic diseases (4). MSCs are able to secrete IL-1Ra (5) and inflammation may be more effectively suppressed by MSCs combining IL-1Ra infusion than separately using IL-1Ra or MSCs (6,7). There have been studies, which have demonstrated that transgenic hematopoietic cells are able to lead to effective IL-1Ra systemic expression (8,9). Based on our previous study regarding transgenic murine BMSCs (mBMSCs) (10), it was hypothesized that a lentivirus-transduced IL-1Ra gene may be capable of stable expression in mBMSCs *in vitro* and the aim of the present study was to investigate this potential.

## Materials and methods

### Experimental animals

The present study was approved by the Independent Ethics Committee of Shanghai Ninth People's Hospital affiliated to Shanghai Jiao Tong University School of Medicine (SJTUSM; Shanghai, China). Normal C57BL/6 (H2^b^) female mice (age, 6-8 weeks; weight, ~25 g) were used in all experiments in order to exclude any effects of immunological interference. The mice were obtained from the Laboratory Animal Center of Shanghai Ninth People's Hospital affiliated to SJTUSM (certificate no. SCXK 2013-009). The handling of the animals was in accordance with the policies of Shanghai Ninth People's Hospital affiliated to SJTUSM and approved by the Animal Experimental Ethics Committee, Shanghai Ninth People's Hospital affiliated to SJTUSM [permit no. HKDL (2013)29].

### Vector construction

A total of 1×10^6^ fresh murine cells were collected and extracted using TRIzol reagent (Invitrogen Life Technologies, Grand Island, NY, USA). The murine cDNA was synthesized by reverse transcriptase M-MLV (Takara Bio Inc., Otsu, Japan). According to *Mus musculus* IL-1Ra, transcript variant 1, mRNA (NCBI Reference Sequence: NM_031167.5 http://www.ncbi.nlm.nih.gov/nuc-core/NM_031167.5, http://www.ncbi.nlm.nih.gov/sites/entrez?db=gene&cmd=search&term=161-81), The primer sequences were as follows: IL-1Ra, forward: 5′-GCTCTAGA(*Xba*I) GCCACC(kozak)ATGGCTTCAGA-GGCAGCC-TG-3′ and reverse: 5′-CGGGATCC(*Bam*HI)CTATTGGTCTTCCTGG -A-AGT-3′. The IL-1Ra cDNA was amplified using a polymerase chain reaction (PCR) machine (Takara Bio, Inc.) and the expression vector was cloned and validated by sequencing (Invitrogen Life Technologies). The plasmid without endo-toxin was extracted using a Qiagen plasmid midi kit (Qiagen, Hilden, Germany).

### Lentivirus production and titration

**Figure 1:**
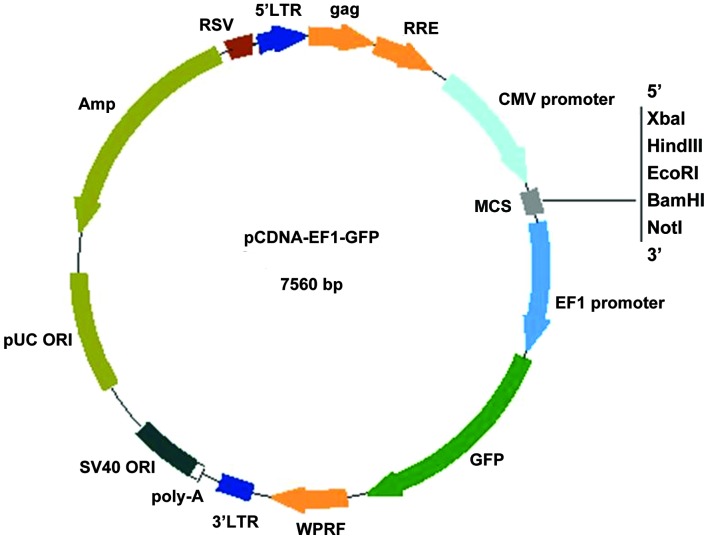
Map of lentiviral vectors.

Using a Lentivector package kit (System Biosciences, Mountain View, CA, USA), the pcDNA-green fluorescent protein (GFP) lentivector was able to be transduced into BMSC genomic DNA and express IL-Ra cDNA constructs. The third generation lentiviral vectors consisted of a three plasmid system and expressed copGFP (Fig. 1). The packaging plasmid, including pPACK-REV, p-PACK-GAG and pVSV-G was transfected into human 293T cells (System Biosciences) using Lipofectamine 2000 (Invitrogen Life Technologies) according to the manufacturer's instructions. The ratio of lentivirus package plasmids mix (500 ng/*μ*l), pcDNA-IL-1Ra plasmid DNA (500 ng/*μ*l) and transfection reagent was mixed at ratio of 20:4:40 *μ*g for the 5×10^5^ cells plated. After an overnight transfection, the cells were cultured in fresh medium consisting of Dulbecco's modified Eagle's medium (DMEM; Gibco-BRL, Carlsbad, CA, USA) and supplemented with 20% fetal bovine serum (FBS; Gibco-BRL). The culture medium was replaced at 16 h post-transfection. The transfection efficiency was assessed at 48 and 72 h post-transfection using a fluorescence microscope (Olympus, Tokyo, Japan) and cell morphology was perceived. The titer of the lentiviral vector was determined via a reverse transcription quantitative PCR (RT-qPCR)-based method while the concentration and purity of DNA and RNA was analyzed by ultraviolet spectrophotometer (Heλiosγ, Thermo Fisher Scientific, Rockford, IL, USA) and protein expression by western blot analysis. The supernatants containing recombinant viruses were collected twice at 48 and 72 h post-transfection. The collected viral supernatants were concentrated via centrifugation (5,000 × g) for 5 min at 4°C and then passed through a 0.45 *μ*m polyvinylidene difluoride (PVDF) membrane filter (Millipore, Billerica, MA, USA) to remove the cellular debris. The concentrated viruses in pellets were incubated at 4°C overnight and then cryopreserved at −70°C.

### Gene transduction of cell lines and titer measurement

Aliquots of unconcentrated viral supernatants of the lentiviral vector were used to determine viral titers by transfer of GFP transgene expression using 293T cells. 293T cells were plated at a density of 1×10^5^ per well (in 2 ml medium) in 6-well plates 1 day prior to transduction. Through use of a gradient dilution method, the titer was attained by the percentage of GFP positive cells of the total cells divided by the dilution time periods. Various dilutions of viral supernatants were administered onto cells in the presence of 8 *μ*g/ml polybrene. After an overnight incubation, fresh medium was added to replace the polybrene and virus-containing medium. For the titer measurements based on GFP transgene expression, transduced 293T cells were cultured for 3 days before the GFP signal was analyzed by fluorescence-activated cell sorting (FACS-Calibur, Becton-Dickinson, Franklin Lakes, NJ, USA). The two assays provided essentially similar values for the titers, measured as transducing units per ml of the supernatants.

### Murine BMSC isolation and expansion

BMSCs were isolated from the bone marrow of mice as previously described (10). Briefly, following euthanasia by cervical dislocation, the bone marrow was flushed from the femoral and tibial compartments with mBMSC growth medium, which consisted of Hepes buffered-DMEM (Gibco-BRL) supplemented with 20% FBS (Gibco-BRL) and 100 U/ml penicillin-G and 100 *μ*g/ml streptomycin sulphate (Gibco-BRL). The recovered suspensions were pooled, counted and plated at a density of 1×10^4^ cells/cm^2^. Non-adherent cells were removed after 24 h and cells were re-fed with mBMSC medium, with additional media changes every 3–4 days. After ~20 days, or once the cell cultures reached confluence, the cells were detached with 0.25% trypsin (w/v)/1 mM EDTA solution (Corning Inc., Danville, VA, USA) and re-plated at 1.5×10^4^ cells/cm^2^, with subsequent passages once they again reached 80% confluence. The mBMSCs between the third and sixth passage were used in the present experiments. Morphological and proliferative characterization of IL-1Ra transduced mBMSCs over 3 weeks were described.

### Flow cytometric analysis

Adherent cells at the third passage (P3) were retrieved by trypsin digestion and aliquots of 1×10^6^ cells were labeled with fluorescein isothiocyanate (FITC), phycoerythin (PE), allophycocyanin (APC) or peridinin-chlorophyll protein complex (PerCP)-conjugated monoclonal antibodies CD29 (β1-integrin), CD34 (non-specific hematopoietic marker), CD44 (phagocytic glycoprotein-1, hyaluronate receptor), CD45 (pan-hematopoietic marker), CD90 (Thy-1) and CD105 (SH2, endoglin) for 30 min at room temperature in the dark. All antibodies were purchased from Becton-Dickinson. Following incubation, the cells were washed in phosphate-buffered saline, centrifuged (700 × g, 5 min) and analyzed using the FACSCalibur (Becton-Dickinson), and data analysis was conducted following grating for the designated population using fluorochrome minus one settings.

### BMSC transfection and GFP reporter gene detection

The P2 logarithmic phase mBMSCs were seeded in 6-well plates at 1.5×10^5^ cells/well, grown overnight and transfected with lentiviral vectors in a minimal volume of medium. The morphology of mBMSCs was assessed under a bright field microscope (NKX41; Olympus, Tokyo, Japan). According to the instructions provided in the Lipofectamine 2000 kit, 10 *μ*l (IL-1Ra) fresh or previously frozen vector supernatants in 3 ml medium were mixed with mBMSCs in the presence of 8 *μ*g/ml polybrene. The lentiviral vector (Lv), Lv.IL-1Ra. copGFP transduced mBMSCs were designated as Lv.IL-1Ra. copGFP/mBMSCs. The cells were cultured for 24 h in DMEM + 20% FBS medium, then for a further 3–4 days in the new medium prior to harvesting for use in the *in vitro* assays. Confluence levels of 80–90% were considered to indicate stable growth. The mBMSCs were harvested for analysis, at the earliest, 96 h post-transfection. For the 28-day trans-gene analysis experiments, the cells were harvested at 7 day intervals, at which point each cell population per well was split, using one half to maintain the cells in culture and the other for GFP expression analysis. An inverted fluorescence microscope (IX71-A12FL/PH; Olympus) was employed to examine the total and GFP-positive cells, as described previously (11). The optimal multiplicity of infection (MOI) was 30. The mBMSCs were divided into the following groups: BMSC + controlLV + IL-1Ra, BMSC + control LV and BMSC alone.

### Viability assessment of mBMSCs

The cell viability of the three groups of mBMSCs were assessed using the cell counting kit (CCK)-8 test kit (Tongren Chemistry, Shanghai, China), respectively. Following the manufacturer's instructions of Trypan blue staining (Sigma-Aldrich, St. Louis, MO, USA) the three groups of P3 and P5 mBMSCs were treated with minimum essential medium-α (Gibco-BRL), 1×10^9^/l cell supernatant and 20 g/l Trypan blue-0.02% EDTA at a ratio of 7.9:0.1:2. The cell percentage, which had been dyed blue was counted with a hemocytometer (Yuejin Medical Instruments, Shanghai, China) and the viability of the three groups of mBMSCs was assessed.

### Growth kinetics analysis

The mBMSC growth was determined using a standard MTT assay (Corning) as described previously (12). Following the third passage, Lv.IL-1Ra. copGFP/mBMSCs were seeded at 5,000 cells per well in 96 plates (Corning) using a hemocytometer (Yuejin Medical Instruments). The cells were detached by treatment with 0.25% trypsin. Between day 1 and 12, each well was administered 20 *μ*l MTT (5 mg/ml) and incubated for 4 h in 5% CO_2_, then stopped using dimethyl sulfoxide and the optical density value was read at a wavelength of 490 nm with a microplate reader.

### IL-Ra mRNA analysis using RT-qPCR

Measurement of IL-Ra mRNA expression in Lv.IL-1Ra.copGFP/mBMSCs was performed using RT-qPCR. RNA was extracted from 5×10^5^ Lv.IL-1Ra. copGFP/mBMSCs from P3, P6 and P9 using TRIzol reagent (Invitrogen Life Technologies), according to the manufacturer's instructions. The murine cDNA was synthesized by reverse transcriptase M-MLV (Takara Bio Inc.). The primers used for the amplification are presented in Table I. RT-qPCR was performed by TP800 using SYBR Premix Ex Taq (Takara Bio Inc.), according to the manufacturer's instructions. β-actin was used as a reference. The real-time PCR conditions were as follows: denaturation at 95°C for 10 sec, 40 cycles at 56°C for 20 sec and 72°C for 20 sec. Dissociation was performed for a melting curve analysis to monitor and avoid non-specific amplification as well as primer dimers. The amplified PCR products were separated on a 1.5% agarose gel using electrophoresis (Takara Bio, Inc.), the bands were visualized using ethidium bromide and images were captured with a UVP imaging system (UVP, Upland, CA, USA). The quantitated mRNA values were normalized against the quantities of HPRT mRNA, and results were administered as -fold induction. PCR analysis was performed at P5.

### Murine IL-1Ra expression using western blot analysis

The sediment of 2×10^7^ P5 mBMSCs was collected via centrifugation (400 × g; 5 min), and whole cellular proteins were extracted using mammalian protein extraction reagent (Pierce Biotechnology, Inc., Rockford, IL, USA). The total cellular protein was quantified using a Pierce Biotechnology protein assay kit (Pierce Biotechnology, Inc.). Sodium dodecyl sulfate- polyacrylamide gel electrophoresis of cellular extracts (50 *μ*g) was performed using 10% acrylamide gels, followed by electrophoretic transfer onto PVDF membranes. The membranes were then probed with the primary antibodies against IL-1ra (cat. no. 5324-1; Santa Cruz Biotechnology, Inc., Santa Cruz, CA, USA) and β-actin (cat. no. 7076; Becton Dickinson) and subsequently with the appropriate secondary antibodies. Positive signals were detected using the enhanced chemiluminescence method (Pierce Biotechnology, Inc.). Equal loading was assessed using β-actin (13).

### Statistical analysis

All data are expressed as the mean ± standard deviation (SD). Comparisons were analyzed using a two-way paired and independent Student's t-test. P<0.05 was considered to indicate a statistically significant difference. Statistical analysis was performed using SPSS 17.0 software (SPSS, Inc., Chicago, IL, USA).

## Results

### Lentiviral vector transduction efficiency

**Figure 2:**
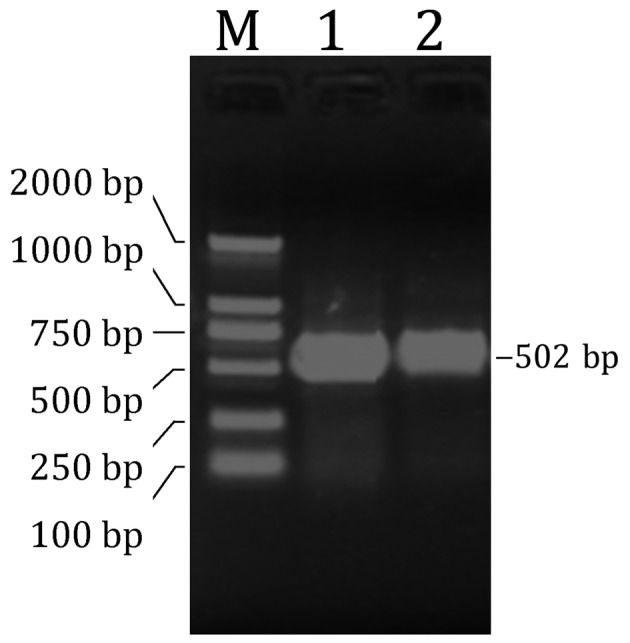
PCR amplified object fragment. The agarose gel electrophoresis revealed that the object band was in the expected position. M, DL2000 DNA marker; 1 and 2, PCR product (502 bp) cDNA. PCR, polymerase chain reaction.

**Figure 3:**
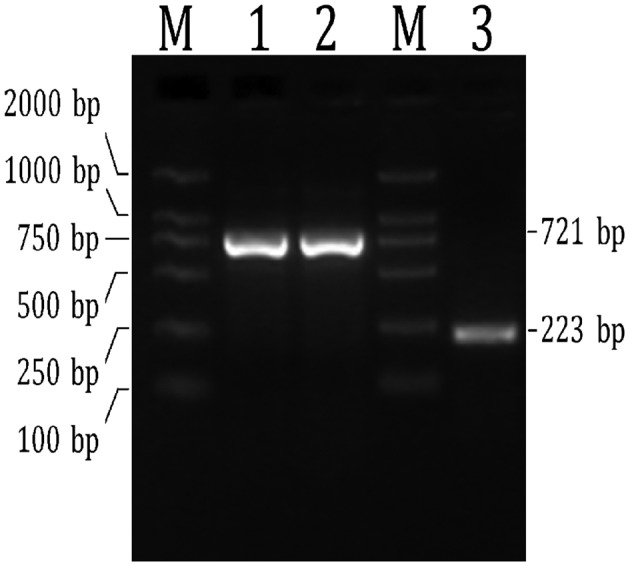
Screening of positive PCR clones. M, DL2000 DNA marker; 1 and 2, PCR product (721 bp) of clone 1 and 2; 3, PCR product of empty carrier. PCR, polymerase chain reaction.

The results of agarose gel by electrophoresis in 100 v for 15 min revealed that the object band was in the correct position of the DNA marker (Fig. 2) and the selected two clones were positive (Fig. 3). The sequencing results revealed that the sequence was the same as the standard sequence with 100% identity (480/480) and 0 gaps (0/480). The transduction efficacy of 293T cells was 85% at 48 h post-transfection. Ultraviolet spectrophotometric analysis of the agarose gel via electrophoresis revealed the purity of the DNA was qualified (data not shown).

### Characteristics of Lv.IL-1Ra.copGFP/mBMSCs

**Figure 4:**
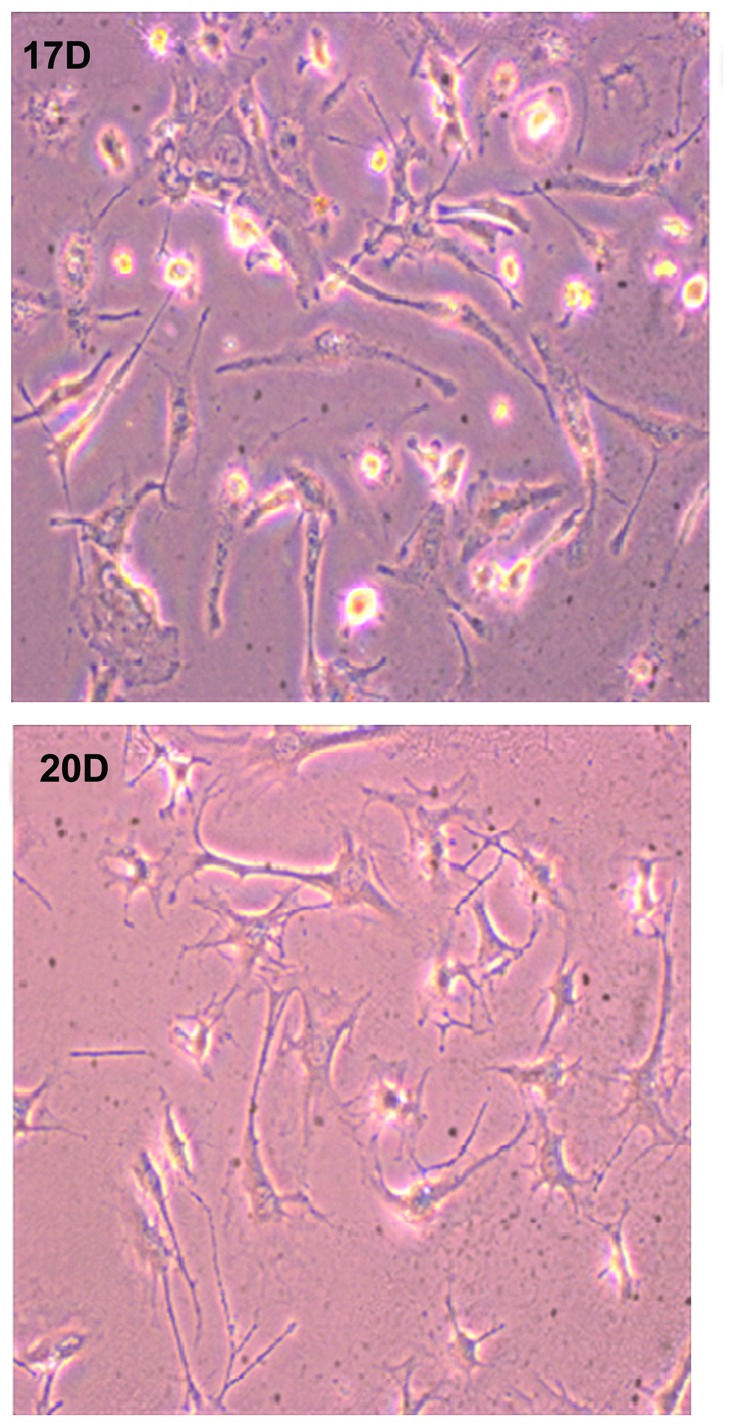
Morphology of murine bone marrow-derived mesenchymal stem cells on different culturing days. Cells began to aggregate on ~17th day (upper) and reached 80% confluence on ~20th day (lower; magnification, ×200).

**Figure 5:**
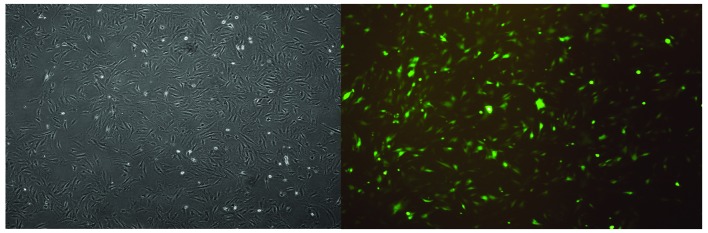
Microscopic evaluation of the Lv.IL-1Ra.copGFP/mBMSCs demonstrating the mBMSCs without transfection (left) and the cells 72 h post-transfection exhibiting 96.53% positivity for GFP (right) at multiplicity of infection of 30 (magnification, ×120). mBMSCs, murine bone marrow-derived mesenchymal stem cells; GFP, green fluorescent protein.

**Figure 6:**
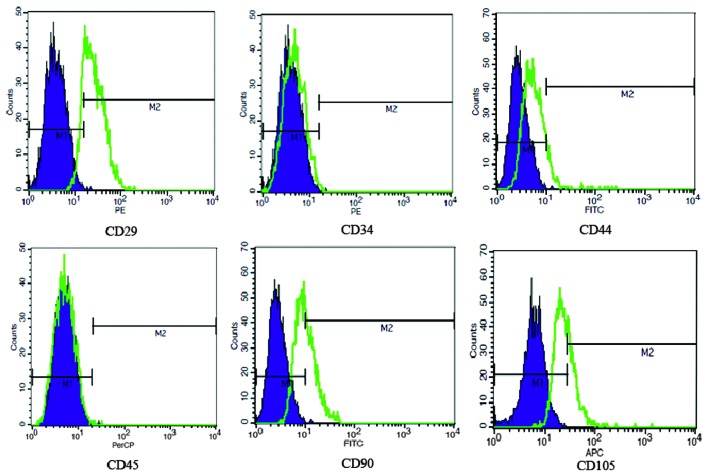
Flow cytometric analysis of epitope characteristics of the murine BMSCs at passage 3. Shaded histogram demonstrating background signal and open histogram demonstrating reactivity with the indicated antibodies. BMSCs, bone marrow-derived mesenchymal stem cells.

The cells began to aggregate after 2 weeks of culture and reached 80% confluence at the 20th day (Fig. 4). The cells proliferated rapidly and were serially passaged twice weekly at a split ratio of 1:4 or 1:3. They did not exhibit any immunogenic damage. The mBMSCs retained a fibroblastic morphology following repeated passages. However, a number of the cells became flattened and polygonal with further subculturing, and cell senescence was usually evident in the culture at P10. The Lv.IL-1Ra.copGFP/mBMSCs were 96.53% positive for GFP at 3 days post-transfection (Fig. 5). The cell surface antigen profile of the mBMSCs was analyzed using flow cytometry and it was identified that >90% of the cells expressed CD29. In addition, they were homogeneously negative for CD34, CD44 and CD45, and the cells were heterogeneous in CD90 and CD105 expression (Fig. 6).

### mBMSC viability and growth kinetics

**Figure 7:**
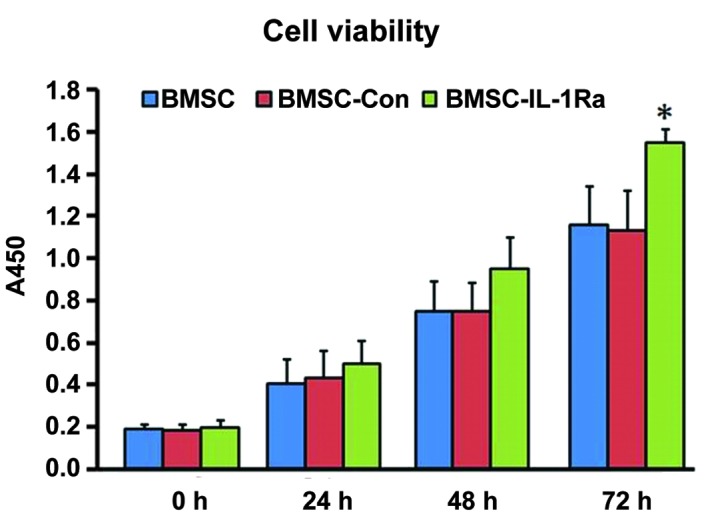
Viability of different mBMSCs using cell counting kit-8 analysis. Cell viability was significantly higher in the BMSC + controlLV + IL-1Ra group at 72 h than in the BMSC and BMSC + controlLV groups (^*^P<0.05, Student's t-test). mBMSCs, murine bone marrow-derived mesenchymal stem cells; LV, lentivirus; IL, interleukin; Con, control.

**Figure 8:**
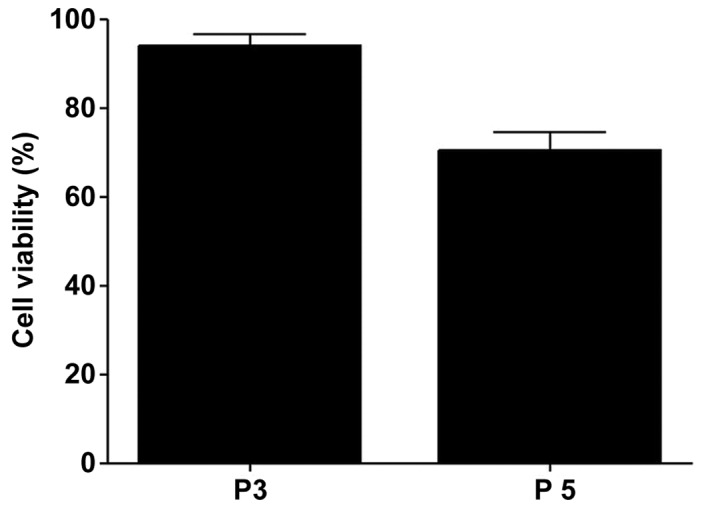
Viability of Lv.IL-1Ra.copGFP/mBMSCs as indicated using Trypan blue staining. It was demonstrated that the cell viability was higher in P3 than in P5. P, passage; mBMSCs, murine bone marrow-derived mesenchymal stem cells; LV, lentivirus; IL, interleukin; GFP, green fluorescent protein.

**Figure 9:**
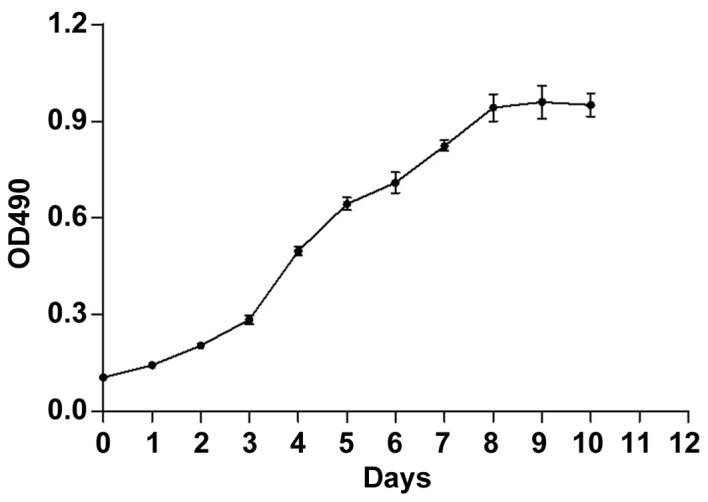
Growth curve of the passage 3 Lv.IL-1Ra.copGFP/mBMSCs. mBMSCs, murine bone marrow-derived mesenchymal stem cells; LV, len-tivirus; IL, interleukin; GFP, green fluorescent protein; OD, optical density.

The CCK-8 kit analysis revealed that the cell viability was significantly higher in the BMSC+ controlLV + IL-1Ra group than in both the BMSC and BMSC + controlLV group at 72 h (t-test, P<0.05; Fig. 7). Trypan blue staining revealed that the cell viability of the three groups was higher at P3 (94.2%) than at P5 (70.6%) *in vitro* (Fig. 8). The growth curve of P3 mBMSCs revealed that the Lv.IL-1Ra.copGFP/mBMSCs were able to grow efficiently up to 11 days (Fig. 9).

### RT-qPCR analysis of Lv.IL-1Ra.copGFP/mBMSCs

**Figure 10:**
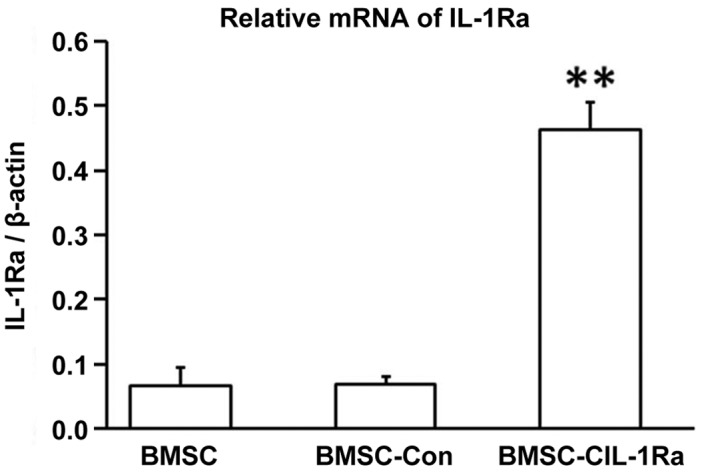
Expression of IL-Ra mRNA demonstrating that the ratio of IL-Ra/β-actin was significantly higher in the BMSC + controlLV + IL-1Ra group than in both the BMSC and BMSC + controlLV group at the fifth passage (^**^P<0.01, Student's t-test). mBMSCs, murine bone marrow-derived mesenchymal stem cells; LV, lentivirus; IL, interleukin; Con, control.

The dissociation temperatures of β-actin and the IL-1Ra gene amplified fragment were 86.8 and 84.9°C, respectively. The ratio of IL-Ra/β-actin was significantly higher in the BMSC + controlLV + -IL-1Ra group (0.46±0.04 SD) than in the BMSC group (0.066±0.28 SD) and the BMSC + controlLV group (0.68±0.12 SD; t-test, both P<0.01; Fig. 10).

### Western blot analysis of Lv.IL-1Ra.copGFP/mBMSCs

**Figure 11:**
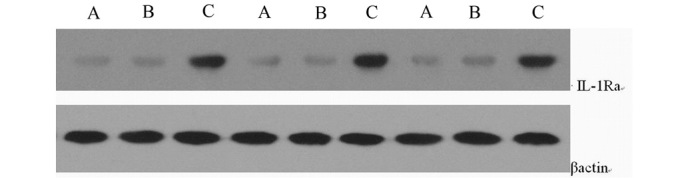
Western blot analysis demonstrating that IL-Ra was expressed effectively only in the BMSC + controlLV + IL-1Ra group (lane C). Lane A, BMSC group; Lane B, BMSC + controlLV group. BMSC, bone marrow-derived mesenchymal stem cell; LV, lentivirus; IL, interleukin.

**Figure 12:**
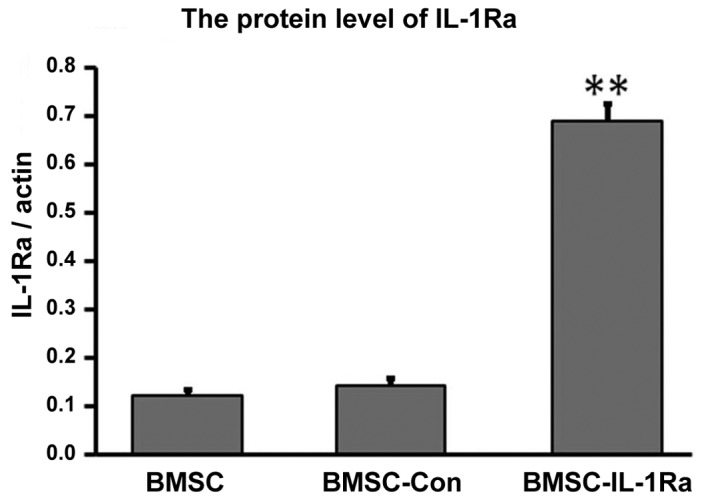
Expression of IL-Ra protein demonstrating that the ratio of IL-Ra/β-actin was significantly higher in both the BMSC + controlLV + IL-1Ra group than in the BMSC and BMSC + controlLV group at the fifth passage (^**^P<0.01, Student's t-test). BMSC, bone marrow-derived mesenchymal stem cell; LV, lentivirus; IL, interleukin; Con, control.

The scanned film revealed that IL-Ra was expressed effectively only in the BMSC + controlLV + -IL-1Ra group (Fig. 11). The ratio of IL-Ra/β-actin was significantly higher in the BMSC + controlLV + -IL-1Ra group (0.69±0.03 SD) than in the BMSC group (0.69±0.03 SD) and the BMSC + controlLV (0.12±0.01 SD) group (t-test, both P<0.01) at P5 (Fig. 12).

## Discussion

The richest source of MSCs in adult organisms is the bone marrow. BMSCs may be easily multiplied *in vitro* and demonstrate capability in secreting a wide range of cytokines, chemokines and growth factors, as well as exhibiting hypo-immunogenic properties (1). MSCs are capable of homing to bone marrow and the sites of tissue repair, inflammation, metabolic disease, regeneration and tumorigenesis (14–17), and do not become tumorigenic following transplantation (5). Preclinical studies and certain clinical trials have revealed that transplantations of BMSCs are able to result in beneficial effects in patients with orthopedic disease (18,19), and also in a model of myocardial infarction (20), corneal injury (21) and peritonitis (22).

The syngeneic mouse does not exhibit immunological rejection, which therefore renders the model superior to other animals in the study of stem cell engraftment. However, the concept is generally accepted that it is difficult to isolate MSCs from murine marrow as the source is insufficient due to the small body mass of the mouse (10). The present study revealed that the primary mBMSCs reached 80% confluence within 20 days, and the cells usually exhibited marked senescence at P10, but not at P15 as was reported by Guo *et al* (23). The authors used compact bones to increase the quantity of stem cells, but cell aggregates usually formed causing difficulties, therefore such mechanical crushing was not a reliable and easy method to isolate and expand mMSCs from mouse BM (24). As the C57BL/6 (H2^b^) mice are syngeneic, based on a previous culture method of our institute (10), samples from several mice were aggregated to collect enough mBMSCs and achieve a successful culture result without observing allogeneic rejection. As the present study demonstrated, the mBMSCs had the capability of proliferation from P2, exhibited peak proliferation at P3 and proliferation declined from P5. This suggested that the mBMSCs had a short proliferation capacity, but sound capability of proliferation at an early stage. In the present study, the cell surface antigen profile of mBMSCs was CD29^+^, CD34^−^, CD44^−^, CD45^−^ and heterogeneous for CD90 and CD105, which were similar to the phenotypic characteristics of an mBMSC population described previously (25–28).

Abundant evidence has indicated an important role for IL-1β in the pathophysiology of various inflammatory conditions, such as rheumatoid arthritis (29), as well as prosthetic aseptic loosening of arthroplasty (30). The IL-1Ra is able to effectively inhibit the biological effects of IL-1 *in vitro* and *in vivo*, but high concentrations are necessary (31). Certain studies have demonstrated that the BMSCs are able to secrete IL-1Ra (5) and had an improved anti-inflammatory effect compared with the exogenous infusion of IL-1Ra (32). Furthermore, combination therapy with IL-1Ra and MSC transplantation is able to promote the restoration and reconstruction of certain serious diseases more efficiently than the use of the two separately (7). When stem cell therapy is combined with a technique involving overexpression, such as gene transfer therapy, IL-1Ra delivered by BMSCs is able to reduce the inflammatory reaction and prevent long-term graft rejection, but the efficiency of adenoviral vector tranducing BMSCs was low and the MOI was as high as 120 (33). In the present study, lentiviral vectors that were more efficient, stable and had improved biosafety compared with adenoviral techniques (2,11,15), were selected. In addition, the MOI was just 30 with mBMSCs 96.53% positive for GFP at 3 days post-transfection. Furthermore, the present study selected the third generation self-inactivating human immunodeficiency virus-1-based VSV-G pseudotyped lentiviral vectors, which consisted of three plasmids and was more efficient than previous studies (2) using copGFP as a reporter gene, which was brighter and had a wider adaptation (pH 4-12.0) than the frequently used enhanced GFP (34). It was hypothesized that these methodological approaches also contributed to the success of the investigation.

In the present study, significant IL-1Ra expression was not detected in primary BMSCs as had been described in previous studies (8,35); however, it was demonstrated that significant exogenous expression of Il-1Ra in lentivirus transduced mBMSCs was maintained for at least 2 weeks, and the Lv.IL-1Ra.copGFP/mBMSCs had a low level of cell death (~5%) and improved cell viability following IL-1Ra transfection (Fig. 7). The overexpression of IL-1Ra in lentivirus transduced mBMSCs may be used to treat early problems associated with arthroplasty or cancer, for which local BMSC infusion is difficult (36,37). The present study was performed *in vitro* and had no inflammatory micromilieu, therefore the mechanism involved with improved growth of mBMSCs following transfection did not involve suppressing inflammatory micromilieu through IL-1Ra (7). It is possible that this is the reason that IL-1Ra were able to nurture BMSCs, however, further investigations are required to clarify the mechanism involved.

In conclusion, numerous factors require clarification before this approach may be transferred to clinical trials, including the infection, immunogenicity and tumorigenesis *in vivo* of Lv.IL-1Ra.copGFP/BMSCs. The results of the present study, demonstrated for the first time, to the best of our knowledge, that lentivirus transduced BMSCs are able to efficiently express the IL-1Ra gene *in vitro* and exhibit improved cell viability following IL-1Ra transfection.

## Figures and Tables

**Table I:** Primers used for reverse transcription quantitative polymerase chain reaction.

Gene	Primer sequence (5′–3′)	Dissociation temperature (°C)	Product (bp)
β-actin	F-CCTGTACGCCAACACAGTGC R-ATACTCCTGCTTGCTGATCC	60	211
IL-1Ra (NM_031167.5)	F-ACCAAATATCAAACTAGAAGAAAA R-CAGAGCGGATGAAGGTAA	56	200

F, forward; R, reverse; IL-1Ra, interleukin-1 receptor antagonist.
